# Na and K Intake from Lunches Served in a Japanese Company Cafeteria and the Estimated Improvement in the Dietary Na/K Ratio Using Low-Na/K Seasonings and Dairy to Prevent Hypertension

**DOI:** 10.3390/nu16101433

**Published:** 2024-05-09

**Authors:** Nagako Okuda, Aya Higashiyama, Kozo Tanno, Yuki Yonekura, Makoto Miura, Hiroshi Kuno, Toru Nakajima, Tomomi Nagahata, Hirokazu Taniguchi, Koki Kosami, Kyoko Kojima, Akira Okayama

**Affiliations:** 1 Division of Applied Life Sciences, Graduate School of Life and Environmental Sciences, Kyoto Prefectural University, Kyoto 606-8522, Japan; nagahata@kpu.ac.jp (T.N.); hirokazu-t@kpu.ac.jp (H.T.); 2 Department of Hygiene, Wakayama Medical University, Wakayama 641-8509, Japan; ayahiga@wakayama-med.ac.jp; 3 Department of Hygiene and Preventive Medicine, Iwate Medical University, Yahaba 028-3694, Japan; ktanno@iwate-med.ac.jp; 4 Department of Nursing Informatics, Graduate School of Nursing Science, St. Luke’s International University, Tokyo 104-0044, Japan; yyonekura@slcn.ac.jp; 5 Collaborative Research Programs of SynCrest Inc., Iwate University, Morioka 020-8550, Japan; mako@iwate-u.ac.jp; 6 Nichinan Kogyo, Co., Ltd., Nikaho 018-0411, Japan; hkuno@kikkonan.co.jp; 7 Research and Development Division, Shoda Shoyu, Co., Ltd., Tatebayashi 374-8510, Japan; 8 Division of Public Health, Center for Community Medicine, Jichi Medical University, Shimotsuke 329-0498, Japan; k.kosami@jichi.ac.jp; 9 The Research Institute of Strategy for Prevention, Tokyo 103-0006, Japanaokayama@jrisp.com (A.O.)

**Keywords:** hypertension, nutrition, sodium, potassium, Japanese, company cafeteria, seasoning, dairy

## Abstract

The excessive intake of sodium (Na) and insufficient intake of potassium (K) are major concerns in the prevention of hypertension. Using low-Na/K seasonings (reducing 25% of the NaCl and adding K salt) may improve the dietary Na/K ratio and help prevent hypertension. To devise an intervention study using low-Na/K seasonings at a company cafeteria, we calculated the Na and K contents of the meals served at the cafeteria and estimated changes in the intakes when suitable low-Na/K seasonings were used. We also considered using milk as a good source of K. We used an ingredient list of a company cafeteria and calculated Na and K contents in each dish. The average amounts of NaCl and K per use were 5.04 g and 718 mg, respectively. Seasonings contributed 70.9% of the NaCl. With the use of low-Na/K seasonings, an estimated reduction in NaCl of 0.8 g/day and an estimated increase in K of 308 mg/day was achieved. With an additional serving (200 mL) of milk, NaCl was reduced by 0.57 g/day and K was increased by 610 mg/day, with an overall decrease in the dietary Na/K ratio from 3.20 to 2.40. The use of low-Na/K seasonings and dairy may improve the dietary Na/K ratio among cafeteria users and help prevent hypertension.

## 1. Introduction

Cardiovascular diseases (CVDs) are the major causes of death in Japan, and the prevention of hypertension, which is a major risk factor for CVDs [1,2], is one of the most important issues in the development of health policy. The prevalence of hypertension in Japan is approximately 10–20% among young adults, but it increases after middle age and exceeds 70% for both men and women over 70 years of age [3,4]. Population-scale strategies must be pursued to prevent hypertension, starting from a younger age.

Among the strategies, salt (sodium chloride, NaCl) reduction and potassium (K) addition may be suitable goals among the modifiable lifestyle factors [5,6,7,8]. In Japan, the average NaCl intake in adults is approximately 10 g/day and has stagnated for the last 10 years, and the average K intake is approximately 2200 mg/day, following a slight downward trend [4]. The World Health Organization recommends a salt reduction target of less than 5 g/day [9] and a K intake target of more than 3510 mg/day [10], both of which substantially deviate from the average intake levels in the Japanese population. Lower urinary Na/K ratios, which have been used as indicators of the dietary Na/K ratio, are associated with lower blood pressure [11,12]. A cohort study in Japan reported lower CVD mortality risk among those with lower dietary Na/K ratios [13]. The international observational INTERSALT study showed that age-related increases in blood pressure are suppressed in populations with a low Na intake and high K intake [14]. Effective measures to reduce Na and increase K (i.e., lowering the Na/K ratio) should be taken for the entire population, including normotensive individuals and the younger generation.

The undesirable trends in Na and K intake in Japan are partially attributed to the increased use of restaurant meals, takeout, and processed foods [15], which externalizes the control of seasonings, usually resulting in a high Na content and a decreased intake of fruits and vegetables (i.e., the major sources of K) [4,16]. These changes in dietary habits may apply to the majority of the working generation in Japan because of an increase in the female employment rate [17], long working hours [18], and a decline in the number of household members [19], which has resulted in a decrease in homemade meals [20,21]. Improving dietary Na/K may be difficult to achieve solely through individual attempts to improve dietary habits. Changes in the food environment, including restaurant and takeout dishes, are needed to decrease the average Na intake and increase K intake. The lack of knowledge regarding the beneficial properties of K among the general population is also a problem. Although target K intake values for preventing hypertension and CVDs are specified in the Dietary Reference Intakes for Japanese [22], education on the hypotensive effect of K and what foods contain it is lacking [23].

K-substituted seasonings in which a certain amount of NaCl is replaced with K salt may be a promising means of lowering dietary Na/K. Furthermore, K has a flavor-enhancing effect and compensates for the decrease in saltiness caused by Na reduction [24]. Several intervention studies using K-substituted salt (low-Na/K salt) have been conducted, and the reported benefits include decreases in blood pressure, CVD incidence and mortality, and medical costs [25,26]. However, the participants of these studies have been limited to older and high-risk individuals, namely residents of senior care facilities [25], poorly controlled hypertensive persons, or those with a history of stroke [26]. The use of low-Na/K seasonings may also have potential benefits in younger populations and may be applicable in many countries; however, suitable low-Na/K seasonings must be considered for practical use because the most frequently used seasonings vary by country. Comparable taste and availability are important factors for the long-term implementation of new seasonings.

According to results from the INTERMAP study in which dietary surveys were conducted in a highly standardized manner to enable quantitative evaluation in four countries, including China and Japan, 75.8% of the Na intake came from salt added during cooking in China [27]. In Japan, Na from salt was rather low (9.5% of the total Na), and high-Na seasonings such as soy sauce (20.0%) and miso (9.7%) were the major Na sources [28]. Moreover, various mixed seasonings (e.g., teriyaki sauce and ramen soup) are often used in restaurants to eliminate cooking procedures and standardize taste. To conduct an intervention study using low-Na/K seasonings in Japan, a variety of seasonings must be considered to determine an effective replacement. If the appropriate low-Na/K seasonings are developed and made available, company cafeterias where the employees eat lunch regularly may be a good setting to study the usability, acceptability, and changes in Na and K intake associated with the use of the seasonings.

To increase K intake, it is also important to promote the consumption of foods that are naturally high in K. While vegetable consumption has been recommended, its consumption is often accompanied by the increased use of high-Na seasonings. Additionally, it is often difficult for company cafeterias to increase the use of vegetables owing to budget, kitchen equipment, and staffing limitations. Notably, milk contains a significant amount of K (~1.5 mg/mL) [29], is available in stable supply, and can be served without cooking or adding Na. It also contains Ca and Mg, which are reported to be effective in preventing hypertension [30,31]. A recent cohort study in Japan reported that milk intake of 7 to 12 cups/week decreased the risk of ischemic stroke in women, compared with an intake of <2 cups/week [32]. However, the average milk consumption is 61.9 g/day [4] and most adults, especially men, do not drink milk regularly. Milk can be a good source of K for serving in cafeterias in Japan, where middle-aged men have lunch regularly.

Herein, we analyzed the Na and K contents in meals at a company cafeteria and considered suitable low-Na/K seasonings that may improve the dietary Na/K ratio and help prevent elevated blood pressure. We estimated the changes in the Na and K intake, and thus the dietary Na/K ratio, of the cafeteria users with the implementation of low-Na/K seasonings, as well as the addition of dairy.

## 2. Materials and Methods

### 2.1. Ingredient List of a Cafeteria

We obtained the menus and the ingredient lists of lunches served at a company cafeteria located in a city in the western part of Japan, and most of the regular users were middle-aged men. The ingredient list was taken from menus served over four weeks (20 business days) in August 2020 and was used to calculate the Na and K contents of the meals. The list specified the names and amounts of the foods used per serving, including foods without added salt, processed foods with added salt (e.g., cured meat and salted vegetables), and seasonings. Seasonings included basic seasonings, such as salt, soy sauce, and miso, and mixed seasonings such as noodle soups and teriyaki sauce. Approximately 210 employees used the cafeteria daily, and the approximate number of servings sold per day was obtained for each meal (i.e., Japanese-style set menu, Western-style set menu, and noodles). We referenced the Na and K contents listed on the manufacturer’s labeling or those from the Standard Tables of Food Composition in Japan [29].

### 2.2. Calculation of the Na and K Contents

The Na and K contents in each dish were calculated. The daily Na and K supply from each food at the cafeteria was calculated by multiplying the Na and K contents by the amount of food used per serving and the average number of servings sold per day prior to the corona disaster. The number of sales in August 2020 in the cafeteria was much lower than usual because telework was encouraged under the corona pandemic, and the sales in August 2020 may have been unrepresentative. The total Na and K supply in August 2020 in the cafeteria was calculated as the sum of the Na and K supply from each food ingredient during that period. The average Na and K intake per use of the cafeteria was calculated based on the total Na and K supply during the study period (20 business days) and the average number of users (n = 210). The Na and K supply was also calculated for each food category (i.e., foods without added salt, foods with added salt, and seasonings). Seasonings were further categorized into basic seasonings and mixed seasonings. The contribution (%) of each food category to the total Na and K supply from each cafeteria meal was calculated. The Na to K ratio (Na/K ratio) was calculated as Na in mmol divided by K in mmol using the atomic weights of Na and K of 23.5 and 39.1, respectively.

### 2.3. Selection of Low-Na/K Seasonings in the Intervention

We intended to reduce the dietary Na/K ratio of the cafeteria lunch through the use of low-Na/K seasonings and dairy. The seasonings are considered low-Na/K if they have 75% of the general Na content and added K almost equivalent to the decrease in Na in the form of K salt (e.g., potassium gluconate and potassium lactate). Five low-Na/K seasonings were commercially available in 2020 (i.e., miso, soy sauce, salt, mentsuyu [soy sauce with fish broth and mirin], and ponzu [soy sauce with citrus juice]). Based on the estimated Na contribution from each seasoning used in the cafeteria menus, we determined the combination of ordinary seasonings that contribute 80% of the NaCl originating from seasonings and should be replaced with low-Na/K seasonings.

### 2.4. Estimated Changes in the Na and K Intake in the Intervention

First, we estimated the changes in Na and K intake with the implementation of low-Na/K seasonings. Since we considered providing dairy to reinforce the increase in K intake, Na and K from the dairy were also included in the calculation. Furthermore, the daily Na and K intake of the cafeteria users was calculated assuming there should be no change in food intake outside of the cafeteria lunch. We assumed an average daily NaCl intake of 11 g (188 mmol) and K intake of 2300 mg (58.8 mmol) for the cafeteria users with an ordinary diet according to the average intake for men in their 40s and 50s reported in the National Health and Nutrition Survey Japan in 2019 [4], considering that most of the cafeteria users were men in their 40s and 50s.

## 3. Results

The estimated averages of the NaCl and K contents of the dishes served in the cafeteria are shown in Table 1. The cafeteria users chose their lunches from several set menus, namely the Japanese or Western dish set (with miso soup, a small plate dish, and plain rice), rice bowl set (with miso soup and a small plate dish), and noodle set (with flavored rice and a small plate dish). The average NaCl and K contents per use of the cafeteria were estimated to be 5–6 g and 600–800 mg, respectively.

The estimated total NaCl and K supply and breakdown into food categories are shown in Table 2. The average amount of NaCl per use of the cafeteria menu was calculated to be 5.04 g (86.2 mmol). The amount of NaCl from foods without added salt was 0.58 g (11.5% of the total NaCl), from foods with added salt, it was 0.89 g (17.7%), and from seasonings, it was 3.57 g (70.8%). The K intake per cafeteria use was 718 mg (18.4 mmol), and the average Na/K ratio (mol/mol) of the cafeteria lunches was calculated to be 4.61.

The seasonings were further categorized into basic seasonings and mixed seasonings, which are generally formulated for noodle soups and dressings. The mixed seasonings consisted of Japanese noodle soup (5 items), Chinese noodle soup (9 items), Japanese dish sauce (5 items), dressing/mayonnaise (12 items), Chinese dish sauce (10 items), tomato ketchup/pasta sauce (7 items), and curry roux (3 items). Among the basic seasonings, miso contributed the most to the Na supply (28.8%), followed by soy sauce (9.3%) and refined salt (2.8%), with a total of the basic seasonings accounting for 42.5% of the total Na in the cafeteria meals. Among the mixed seasonings, noodle soups for Japanese-style noodles and Chinese noodles accounted for 10.1% and 5.8% of the total Na, respectively. Other mixed seasonings (46 items) accounted for 12.5% of the total Na.

To achieve a substantial reduction in Na/K, we attempted to select low-Na/K seasonings that can replace 80% of the Na in the original seasonings. According to the analysis of the menus, the Na in the basic seasonings, soup for Japanese noodles, and soup for Chinese noodles accounted for 58.4% of the total Na and 82.5% of the Na from all seasonings. Thus, we focused on developing low-Na/K noodle soups (i.e., Japanese noodle soup [for udon and soba] and four flavors of Chinese noodle [ramen] soup [pork bone, pork bone and soy sauce, miso, and white broth]). For Japanese dish sauces, such as teriyaki sauce, low-Na/K soy sauces, salts, and sweeteners (sugar, mirin) were intended to be used. For other seasonings with lower contributions to the total Na supply, the existing seasonings were used as normal.

The estimated changes in Na and K with the use of low-Na/K seasonings (basic seasonings and noodle soups) and dairy are shown in Table 3. It was estimated that a reduction in NaCl of 0.8 g and an increase in K of 301 mg per use of the cafeteria would be achieved with the use of low-Na/K seasonings. With an additional 200 mL of regular milk, which contains 0.22 g of NaCl and 310 mg of K, a reduction in NaCl of 0.57 g and an increase in K of 611 mg was estimated. For the estimation, the Na and K contents for regular (normal fat) milk were used because only 15.1% of people who drank milk regularly answered that they usually have low-fat milk in a survey in 2016 [33], and the average consumption was higher for milk (61.9 g/day) than for yogurt (37.0 g/day) in 2019 [4].

Furthermore, we estimated the changes in the daily dietary intake of Na, K, and Na/K. According to the estimates showing that cafeteria users consumed 5.04 g of NaCl and 718 mg of K from lunch, we assumed that they consumed 5.96 g of NaCl and 1583 mg of K from foods outside the cafeteria, which accounted for the differences from the average total intake of NaCl (11 g, 188 mmol) and K (2300 mg, 58.8 mmol) for average middle-aged Japanese men [4]. The daily dietary Na/K ratio was calculated to be 3.20. With the use of low-Na/K seasonings in the cafeteria lunch, the total daily NaCl and K intake was estimated to be 10.2 g and 2601 mg, respectively, and the dietary Na/K ratio decreased to 2.63. With an additional pack of milk, the Na/K ratio decreased further to 2.40, achieving a 0.80 decrease in dietary Na/K. Changes in dietary Na/K were monitored by measuring the Na and K concentrations of spot urine during the intervention. Since nearly all dietary Na is excreted in the urine, whereas ~80% of K is excreted, the difference in the urinary Na/K ratio with the use of low-Na/K seasonings and dairy was estimated to be approximately 1.

## 4. Discussion

From the analyses of the ingredient list for lunches served at a Japanese company cafeteria, we estimated that the users consumed 5.04 g of NaCl and 718 mg of K per use of the cafeteria, on average resulting in a Na/K ratio of 4.61 mmol/mmol. By replacing the ordinary basic seasonings and noodle soup seasonings with low-Na/K types, an estimated reduction in NaCl of 0.80 g and an estimated increase in K of 301 mg were determined. With an additional supplement of milk (200 mL/day), the reduction in NaCl became 0.57 g, with an increase in K of 610 mg. Assuming that the diets outside the cafeteria remain the same, a decrease of 0.80 in the dietary Na/K ratio may be expected for the cafeteria users. To the best of our knowledge, this is the first study in which lunch menus of a Japanese company cafeteria were analyzed to examine the Na and K supply and estimate changes with the use of low-Na/K seasonings and dairy. For those who eat three meals a day and work five days a week, the cafeteria lunches account for approximately 24% of their total nutrition (=1/3 × 5/7). Thus, employers can help promote employees’ health by improving the nutrition of the cafeteria food.

The health benefits of sodium reduction have been examined in many aspects [34,35]. In addition to the associations with lower blood pressure and the risk of CVD, it has also been reported to be associated with reduced renal dysfunction [36] and reduced risk of osteoporosis [37]. High salt intake has also been reported to be associated with gut microbiota [38]. Moreover, an intervention study in untreated hypertensive patients reported that serum concentrations of short-chain fatty acids (SCFAs) of gut microbial origin increased significantly in the modest sodium reduction group, and the increase in SCFAs was associated with lower blood pressure [39]. On the other hand, in a randomized controlled trial in which appropriately treated heart failure patients were enrolled, strict sodium restriction showed no difference in outcomes including admission and all-cause death compared to controls [40]. The effects of sodium restriction may vary depending on the characteristics of population, and the degree and duration of sodium restriction. It is desirable to test the effects in a variety of settings and intervention methods including use of substituted salt.

The effect of reducing the dietary Na content and increasing K on blood pressure may be more pronounced in older hypertensives because of their reduced regulatory capacity [35,41,42]. Although the immediate effects of Na/K reduction are unlikely to be apparent in young normotensives, continued dietary Na/K low levels from youth may suppress age-related increases in blood pressure [14]. A decrease in dietary Na/K in the younger generation may reduce the number of hypertensive individuals in the future, lowering the incidence of diseases attributable to hypertension (i.e., stroke, heart disease, and chronic kidney disease [CKD]). In our previous study, we surveyed company employees about their knowledge of hypertension prevention [23]. In the survey, 30.6% of the men and 18.3% of the women reported that they had hypertension, and 38.1% of the men and 54.9% of the women stated that they were interested in salt reduction. From our current analyses, many of the people concerned about high salt intake probably eat lunches with 5 g of NaCl, but they do not have much choice. The use of low-Na/K seasonings is one way to provide a low-Na/K option for those interested in salt reduction. What an individual eats and how he/she is nourished are highly influenced by the food environment in which they live. Even if individuals are knowledgeable and sufficiently motivated, it is difficult to improve their nutrition unless they are provided with suitable foods that can be used continuously in their regular diet. In addition to encouraging salt reduction and K intake from appropriate foods, suitable low-Na/K seasonings may be useful in improving dietary Na/K at the individual level, as well as the population level.

The renal capacity of K excretion is abundant in healthy individuals and there is no need to restrict K intake, but in patients with CKD and decreased K excretion, it may be necessary to restrict K intake to avoid adverse outcomes, including hyperkalemia. However, there is not much evidence to support K restriction in CKD patients. A systematic review of follow-up studies concerning dietary K intake and CKD progression in late-stage CKD patients did not find that higher K intake is associated with higher mortality [43]. In an intervention study using a K-based salt substitute in CVD high-risk participants, the incidence of serious adverse events attributed to hyperkalemia was not significantly different between the groups using K salt and regular salt [26]. The updated KDOQI clinical practice guideline for nutrition in CKD [44] only recommends people “to adjust dietary potassium intake to maintain serum potassium within the normal range,” and no numerical target value for K intake is given. The Japanese Society of Nephrology suggests moderate K restriction (<2000 mg/day) for those with CKD stage G3 and more severe restriction (<1500 mg/day) for stages G4/G5, after considering serum K levels and the use of medications [45]. Patients with CKD who have substantially impaired renal function are often on K-retaining medications, and serum K levels should be monitored to avoid complications such as arrhythmias caused by hyperkalemia. Overall, the use of low-Na/K seasonings in the company cafeteria is unlikely to cause problems associated with the additional K intake.

In Japan, the provision of meals in company cafeterias is part of the welfare program implemented by the employer to promote the health and well-being of employees [46]. Employers setting up cafeterias are supposed to provide appropriate nutritional management that meets the needs of the working generation [47], and preventing hypertension should be considered as part of this goal. The dietary Na/K ratio can easily be improved if low-Na/K seasonings are used in place of ordinary seasonings in the cafeteria kitchen without changing the cooking process. If the cafeteria users consider the meals cooked with low-Na/K seasonings to be as good as ever, it is possible to offer the meals continuously. To verify this, we held a tasting session and served sample dishes using prototypes of the new low-Na/K seasonings. In addition to three flavors of low-Na/K Chinese noodles, two dishes with low-Na/K soy sauce and miso were cooked in the cafeteria kitchen, and ten volunteer employees tasted them. They were asked to rate how salty the food tasted, and the overall opinion was favorable. None of them rated any of the dishes as “lightly salted” or “not tasty.” Thus, the appropriate use of low-Na/K seasonings is effective in improving the Na/K ratio of cafeteria meals.

There were some limitations in this study. First, our estimates were based on data from one cafeteria and may have limited generalizability. Furthermore, the users may not eat the whole servings. Finally, because the amount of seasoning used is ultimately deter-mined in the kitchen by the taste test, the actual amount of seasoning may have been different from our estimations based on the ingredient list and the Standard Tables of Food Composition in Japan 2020, 8th edition [29], or the manufacturer’s labeling. The expected change in Na/K ratio was calculated based on the averages of Na and K intakes of middle-aged men from the National Health and Nutrition Survey Japan, and not using the results of urinalysis or other dietary survey in the employees.

## 5. Conclusions

By replacing basic seasonings and noodle soups with low-Na/K seasonings in lunches served at a company cafeteria, an estimated reduction in NaCl of 0.8 g and an increase in K of 301 mg was achieved for the average meal. The use of low-Na/K seasonings and a pack of milk can reduce the daily NaCl intake by 0.57 g, increase the daily K intake by 610 mg, and decrease the dietary Na/K ratio by 0.80 for regular cafeteria users. The use of low-Na/K seasonings and dairy may help improve the dietary Na/K ratio among cafeteria users and help prevent hypertension.

## Figures and Tables

**Table 1:** NaCl and K contents of the dishes served in a company cafeteria in August 2020 (20 business days).

Type of Dishes	Type of Dish Served Each Day	NaCl (g/Serving)		K (mg/Serving)	
		Mean	(SD)	Mean	(SD)
Main dishes for Japanese set menu	1	2.15	(1.15)	485	(159)
Main dishes for Western set menu	1	1.66	(0.59)	479	(155)
Miso soup	1	1.96	(0.74)	94	(46)
Plain rice (white rice, black rice mixed)	2	0	(0)	84	(6)
Rice bowl dishes, curried rice	1	2.61	(1.04)	446	(98)
Noodles	1	4.77	(1.75)	312	(102)
Flavored rice for noodle set	1	0.86	(0.95)	100	(82)
Small plate dishes	5	0.61	(0.42)	124	(69)

SD, standard deviation.

**Table 2:** Estimated sources of NaCl (g) and K (mg) in meals provided at a company cafeteria averaged over 20 business days in August 2020.

		NaCl (g) per Use (%)		K (mg) per Use (%)		Na/K Ratio (mmol/mmol)
Foods without added salt		0.58	(11.5)	490	(68.3)	0.79
Foods with added salt		0.89	(17.7)	136	(19.0)	4.35
Seasonings						
Basic seasonings						
	Miso	1.45	(28.8)	43	(6.0)	22.36
	Soy sauce	0.47	(9.3)	12	(1.6)	26.42
	Salt	0.14	(2.8)	0	(0)	-
	Ponzu (soy sauce with citrus juice)	0.06	(1.2)	2	(0.3)	16.98
	Mentsuyu (soy sauce with fish broth and mirin)	0.05	(1.0)	1	(0.1)	62.78
	Total of the basic seasonings	2.14	(42.5)	58	(8.1)	24.97
Mixed seasonings						
	Soup for Japanese noodles (5 items)	0.51	(10.1)	7	(0.9)	55.07
	Soup for Chinese noodles (9 items)	0.29	(5.8)	4	(0.6)	44.26
	Dressing, mayonnaise (12 items)	0.15	(3.0)	2	(0.3)	49.67
	Mixed sauce for Japanese dish (5 items)	0.15	(3.0)	3	(0.5)	28.61
	Mixed sauce for Chinese dish (10 items)	0.11	(2.2)	2	(0.3)	60.69
	Tomato ketchup, pasta sauce (7 items)	0.09	(1.8)	10	(1.4)	5.62
	Curry roux (3 items)	0.07	(1.4)	2	(0.2)	25.61
	Japanese dressing (4 items)	0.04	(0.8)	1	(0.1)	30.87
	Granular bouillon (5 items)	0.03	(0.5)	0	(0)	90.34
	Total of the mixed seasonings	1.43	(28.4)	25	(3.5)	39.22
Total of the seasonings		3.57	(70.8)	82	(11.4)	29.07
Total		5.04	(100)	718	(100)	4.61

**Table 3:** Breakdown of the estimated average NaCl (g) and K (mg) intake per use of the cafeteria for the ordinary diet (OD), low-Na/K diet, and low-Na/K diet plus a pack (200 mL) of milk.

			OD	Low-Na/K Seasonings (Diff. from OD)		Low-Na/K Seasonings Plus Milk (Diff. from OD)	
NaCl intake (g/use of the cafeteria)							
	Cafeteria lunch		5.04	4.25	(−0.80)	4.47	(−0.57)
		Foods without added salt	0.58	0.58	(0)	0.58	(0)
		Foods with added salt	0.89	0.89	(0)	0.89	(0)
		Seasonings, replaced with a low-Na/K type	3.09	2.29	(−0.80)	2.29	(−0.80)
		Seasonings, not replaced	0.49	0.49	(0)	0.49	(0)
		Milk (normal fat, 200 mL)	0	0	(0)	0.22	(0.22)
	Foods outside the cafeteria		5.96	5.96	(0)	5.96	(0)
	Total in a day		11.00	10.20	(−0.80)	10.43	(−0.57)
K intake (mg/use of the cafeteria)							
	Cafeteria lunch		718	1019	(301)	1328	(610)
		Foods without added salt	504	504	(0)	504	(0)
		Foods with added salt	125	125	(0)	125	(0)
		Seasonings, replaced with a low-Na/K type	70	371	(301)	371	(301)
		Seasonings, not replaced	20	20	(0)	20	(0)
		Milk (normal fat, 200 mL)	0	0	(0)	309	(309)
	Foods outside cafeteria		1583	1583	(0)	1583	(0)
	Total in a day		2300	2601	(301)	2910	(610)
Dietary Na/K ratio (mmol/mmol)							
	Cafeteria lunch		4.61	2.89	(−1.7)	2.22	(−2.39)
	Foods outside cafeteria		2.55	2.55	(0)	2.55	(0)
	Total of a day		3.20	2.63	(−0.57)	2.40	(−0.80)
Urinary Na/K ratio (mmol/mmol)			4.00	3.29	(−0.71)	3.00	(−1.00)

## Data Availability

The original contributions presented in the study are included in the article, further inquiries can be directed to the corresponding author.
